# COVID‐19 vaccination in Nigeria: Challenges and recommendations for future vaccination initiatives

**DOI:** 10.1002/puh2.57

**Published:** 2023-01-12

**Authors:** Taiwo Oluwaseun Sokunbi, Abdmateen Temitope Oluyedun, Emmanuel Ayomide Adegboye, Glory Peace Oluwatomisin, Abdulmumin Damilola Ibrahim

**Affiliations:** ^1^ Faculty of Pharmacy Obafemi Awolowo University Ile‐Ife Nigeria; ^2^ Faculty of Pharmaceutical Sciences University of Ilorin Ilorin Nigeria

**Keywords:** Africa, COVID‐19, Nigeria, pandemic, vaccination

## Abstract

The COVID‐19 vaccination program is the most extensive vaccination program held in Nigeria to date. As of September 21, 2022, more than 31 million people have been fully vaccinated which made up 15% of the entire population. Following the global COVID‐19 vaccination goal, Nigeria was expected to vaccinate 40% of its population in 2021 and reach the 70% vaccination threshold before the end of 2022. Currently, Nigeria is nowhere near the global vaccination goal due to various challenges encountered by the program. Challenges such as distrust in government, poor cold‐chain management, and poor communication during the onset of the program all contributed to the inability to attain the set goal. With the pledge of Global Alliance for Vaccines and Immunizations to extend the malaria vaccination program to other Sub‐Saharan African countries after the success of the first dose vaccination in Ghana, Kenya, and Malawi and the recent reemergence of monkeypox virus in Africa which also requires vaccination to curtail its spread, it is important that Nigeria, in preparation for these vaccination programs, learn from some of the challenges that the COVID‐19 vaccination program encountered and take actions to ensure greater success in future vaccination programs in the country. This paper aims to give an overview of the COVID‐19 vaccination program in Nigeria, highlight the challenges encountered, and provide recommendations for better future vaccination initiatives in the country.

## INTRODUCTION

Nigeria confirmed the first novel coronavirus disease 2019 (COVID‐19) case in February 2020 [1]. The disease that was first reported in Wuhan province of China spread all over the world and was finally declared a pandemic by the World Health Organization (WHO). The novel disease has had huge socioeconomic impacts on people and affected health systems and the global economy to the extent that many countries went into economic recession [1]. Certain responses were activated in an attempt to curb the spread of the virus nationwide, such as border closure, restriction of movement and lockdown, social gathering restriction, and enforcement of the use of noise masks and hand washing [2].

In the midst of a pandemic, vaccination is the most effective public health intervention in curtailing the spread of the disease. It is known to be crucial in disease elimination, and disease prevention, and also effective for the reduction of disease mortality rate as well as giving herd immunity to an unvaccinated population [1]. The COVID‐19 vaccination program began in Nigeria in March 2021, and in alignment with the global COVID‐19 eradication goal, Nigeria was expected to vaccinate 40% of its population at the end of 2021 and attain a 70% vaccination threshold in 2022 [3]. However, as of September 21, 2022, only 15% of the country's population have been fully vaccinated [4]. This paper aims to give an overview of the COVID‐19 vaccination program in Nigeria, highlight the challenges encountered, and provide recommendations for better future vaccination initiatives in the country.

## COVID‐19 VACCINATION IN NIGERIA

COVID‐19 vaccination is the largest vaccination program held in Nigeria. The first COVID‐19 vaccine of about 4 million doses of the AstraZeneca/Oxford vaccine arrived in the country on March 2, 2021, through the COVID‐19 Vaccine Global Access (COVAX) facility, a partnership between Coalition between Epidemic Preparedness Innovations (CEPI), Global Alliance for Vaccines and Immunizations (GAVI), United Nations Children's Fund (UNICEF), and WHO. This is an attempt to ensure equitable distribution of the COVID‐19 vaccines globally. Immediately after the arrival of the vaccine, vaccination began with the frontline health workers as they are one of the most prioritized groups to receive the COVID‐19 vaccine [5]. Since the arrival of the first wave of vaccines, the country has received more than 67 million doses of COVID‐19 vaccines, out of which 31.1 million people have been fully vaccinated [4]. Currently, apart from Oxford vaccine, six (6) more vaccines have been approved for use of in Nigeria. These include: Moderna vaccine, Pfizer/BioNTech, Gamaleya, Janssen (Johnson & Johnson), Serum Institute of India vaccine, and Sinopharm (Beijing) [6].

For a vaccination program to be effective there must be a high acceptance rate of the program. In Nigeria, the rate at which people are willing to take the vaccine is low [7]. In December 2021, due to poor vaccine uptake in Nigeria, about 1 million AstraZeneca vaccines became expired [7]. A review aimed at assessing the acceptance rate of the COVID‐19 vaccine among adults in six geopolitical zones of the country found the acceptance rate to be between the ranges of 20%–58.2% [7]. The study also identified conspiracy theories, side effects, and misinformation spread on social media as some of the reasons for the unwillingness toward the vaccine [7]. Among those who took the vaccine, a study stated that education, religion, and prior COVID‐19 diagnoses all had a significant impact on the vaccine uptake [8]. These factors can be linked to where the trust of the population lies. It is therefore no surprise that Nigeria is nowhere near the 70% vaccination threshold before the end of 2022.

## CHALLENGES OF COVID‐19

One of the challenges of COVID‐19 vaccination in Nigeria is poor communication at the onset of the program. At the onset of the COVID‐19 vaccination program in Nigeria, there was a problem of inadequate publicity to create awareness of the availability of vaccines. Information on the vaccination rollout was not accessible to many people in their local languages, so comprehension of the benefits of the vaccine became difficult. Hence, the safety of the vaccine was in doubt. Moreover, at the beginning of the program, people were expected to first make an appointment online to be able to get vaccinated. The inability of many Nigerians to access the internet and lack of technical know‐how of digital technology resulted in a low turn up for the vaccine. These affected the country's ability to vaccinate at least 40% of its population at the end of 2021 [9].

Poor cold‐chain system and infrastructure also posed threats to the success of the vaccination program. Some of the vaccines developed for COVID‐19 require extremely cold storage to increase their shelf‐life. Moderna and Pfizer vaccines require being stored at 2–8°C and −70°C temperature, respectively, before distribution and therefore require vaccine cold‐chain management for their effective storage and transportation. With the burden of COVID‐19 in Nigeria, the current cold‐chain storage capacity of 201 m^2^ in the country in comparison with the 672 m^2^ capacity required was insufficient for an optimal vaccination program. In addition to this, the inadequate capabilities of the cold‐chain workers to understand the proper functioning of the system posed a threat to the efficacy and efficiency of the cold‐chain system and consequently impacted the overall outcome of the vaccination program [10].

The COVID‐19 vaccination program acceptance rate in Nigeria is slow. The unwillingness of the public to take the vaccine stems from the lack of trust in the government [7]. In a study conducted by Ryota Sato on vaccine hesitancy and trust in government in Nigeria, the study revealed that the low vaccination coverage in the country is a result of distrust in government [11]. This distrust comes mostly from the rural dwellers who believed that the vaccine is not safe and harmful to them because it came from the government [11].

## RECOMMENDATIONS FOR FUTURE VACCINATION INITIATIVES

With the pledge of GAVI to extend the malaria vaccination program to other sub‐Saharan African countries after the success of the first dose vaccination in Ghana, Kenya, and Malawi [12], and the recent reemergence of monkeypox virus in Africa which also requires vaccination to curtail its spread, it is important that Nigeria, in preparation for these vaccination programs, learn from some of the challenges that the COVID‐19 vaccination program encountered and take actions toward more successful future vaccination programs in the country.

The ongoing COVID‐19 vaccination has exposed the weaknesses of the country's cold‐chain systems for vaccine storage, distribution, and efficient administration. Going forward, the government should reorganize the vaccines supply chain into a system that fosters fast availability of vaccines to all states, increase the storage capacity for effective vaccine storage to protect vaccines potency, and heavily invest in technologies such as solar‐powered refrigerators for efficient vaccines cooling as well as in the training of vaccinators and vaccine technicians [9]. When the cold‐chain system is sufficient and properly maintained, there will be minimal wastage of vaccines. Although Nigeria has some established refrigerated warehouses and solar powered refrigerators, but these are still insufficient to cater for its population [13].

Additionally, to increase public trust in vaccination programs in Nigeria, the government should first earn the trust of the people through periodic, clear, and transparent communication then encourage local production of vaccines through investment in research and development. When vaccines are locally produced, the challenges of low vaccine acceptance, as well as vaccine hesitancy in the country will be significantly reduced. People will feel safer taking the vaccine and there will be fewer conspiracy theories around the vaccine. Furthermore, the government should not wait until the malaria and monkeypox vaccines arrive in the country to create vaccine awareness. Vaccine education programs should be integrated as part of public health interventions in the country to educate people in their local languages to debunk myths about vaccines and tell them about the importance and benefits of vaccines to individual health. This should be regular program not until there is need for it. Moreover, to solve the problem of poor communication, health‐care providers should take it upon themselves to build effective communication channels with the general populace, most especially to the people living in the rural areas to encourage timely dissemination of health‐care information to these people.

## CONCLUSION

The COVID‐19 vaccination program has shown some of the weaknesses in Nigeria's vaccination system. It is high time the Nigerian government and policymakers revisit some of the challenges encountered by the program in preparation for better future vaccination programs in the country. Doing this will ensure the optimal efficiency of subsequent vaccination programs in Nigeria.

## AUTHOR CONTRIBUTIONS

*Conceptualization; writing, and reviewing the first and final draft the study*: Taiwo Oluwaseun Sokunbi. *Writing the first draft*: Abdmateen Temitope Oluyedun. *Writing the first draft*: Emmanuel Ayomide Adegboye. *Writing the first draft*: Glory Peace Oluwatomisin. *Reviewing the paper*: Abdulmumin Damilola Ibrahim.

## CONFLICT OF INTEREST

The authors declare no conflict of interest.

## Data Availability

Data sharing is not applicable to this article as no new data were created or analyzed in this study.
